# Short-term symptom improvement in infants with suspected cow’s milk protein allergy using amino acid formula: a prospective cohort analysis

**DOI:** 10.3389/fnut.2023.1208334

**Published:** 2023-06-20

**Authors:** Michael J. Wilsey, Jessica V. Baran, Luke Lamos, Jesse Beacker, Jared Florio, Lea Oliveros, Panida Sriaroon, Jerry M. Brown, Jon A. Vanderhoof

**Affiliations:** ^1^Department of Pediatrics, University of South Florida Morsani College of Medicine, Tampa, FL, United States; ^2^Pediatric Gastroenterology, Hepatology, and Nutrition of Florida, St. Petersburg, FL, United States; ^3^Office of Medical Education, Florida Atlantic University Charles E. Schmidt College of Medicine, Boca Raton, FL, United States; ^4^Department of Pediatrics, Division of Allergy and Immunology, University of South Florida Morsani College of Medicine, Tampa, FL, United States; ^5^Department of Gastroenterology Hepatology and Nutrition, Boys Town Hospital, Boys Town, NE, United States

**Keywords:** cow’s milk protein allergy (CMPA), milk hypersensitivity, eczema, infant nutrition, amino acid formula, ZSMoments

## Abstract

**Background:**

Cow’s milk protein allergy (CMPA) occurs commonly in infants. While the long-term efficacy of amino acid formulas for managing CMPA is well-established, there is limited data on the short-term symptom improvement of using amino acid formula (AAF).

**Objective:**

This study aimed to determine the short-term effects of managing suspected CMPA in infants aged 6 months and under using a commercial AAF.

**Methods:**

Healthcare providers who treated infants with suspected CMPA aged 6 months or younger (*n* = 104) provided de-identified survey data in this prospective study. Healthcare providers scored symptoms for severity from 0 to 3 (none, low, moderate, severe) before using a commercial AAF at Visit 1 and at Visit 2 (3–6 weeks later).

**Results:**

Gastrointestinal (94%), skin (87%), respiratory (86%), and uncategorized symptoms (89%) improved from AAF initiation, and these findings were consistent across different follow-up visit durations.

**Conclusion:**

This study is the most extensive prospective analysis conducted in the United States examining the short-term change in suspected CMPA symptoms using an AAF. These findings suggest that AAF may decrease the severity of suspected CMPA symptoms in infants 6 months or younger, often by the next follow-up visit. Further randomized controlled trials are required to confirm these initial findings.

## 1. Introduction

Food allergy is an immunological reaction caused by various food antigens. The incidence of food allergies has increased in the past 20 years, specifically in industrialized countries (1). One of the most common pediatric food allergens is cow’s milk protein (2). Cow’s milk protein allergy (CMPA), an immunological consequence of cow’s milk protein typically present in breast and cow’s milk, occurs when a child’s immune system recognizes cow’s milk protein as a foreign invader and initiates an immune response (3). CMPA prevalence depends on the population and age group affected and is between 1.9 and 4.9% (2, 4). Most children with CMPA are <1 year old, and some resolve CMPA within the first year of life (2, 5). CMPA has become a significant matter of public health for physicians and parents due to its high prevalence, risk of severe complications, and impact on the quality of life for families (2).

Clinicians commonly classify CMPA into two types: Immunoglobulin-E (IgE) mediated and non-IgE mediated. These immune types are further characterized as immediate hypersensitivity and delayed-type (cell-mediated) hypersensitivity reactions, respectively (2, 4, 6–8). The non-IgE type typically presents hours to days after ingesting cow’s milk protein (2–4, 9–11). The most common symptoms of cow’s milk protein allergy in infants include gastrointestinal symptoms such as diarrhea, vomiting, abdominal pain, and hematochezia (2, 5, 12, 13). Infants may also experience skin symptoms such as eczema and acute urticaria, as well as respiratory symptoms such as coughing, wheezing, and runny nose (2, 5, 12, 13). However, in some cases, CMPA can cause severe symptoms, including anaphylaxis (2, 5, 12–15). Symptom severity and frequency may vary widely between patients as mechanisms of CMPA and tissue location affected vary between patients (2, 13). CMPA in infants can lead to various complications. Infants with CMPA may experience weight loss and failure to thrive if left untreated. Additionally, long-term complications of CMPA may include iron deficiency anemia, developmental delays, behavioral disorders, and an increased risk of other allergic conditions, such as allergic gastroenteropathy, asthma, and eczema.an Therefore, early diagnosis and management of CMPA are crucial to avoid such complications (5, 16).

Cow’s milk protein allergy is typically diagnosed clinically by pediatric healthcare providers (HCPs) (2, 5, 9, 10). Diagnosis involves an assessment of clinical symptoms, an allergy-focused history, physical examination, and resolution of symptoms after eliminating cow’s milk protein from the diet (2, 5, 9, 10). CMPA presents with a wide range and severity of symptoms, often resembling other diseases, therefore increasing the difficulty in clinical definitive diagnosis (2). Additionally, the lack of pathognomonic symptoms of CMPA further complicates clinical diagnosis. The gold standard diagnosis of CMPA is by using an elimination diet in which cow milk protein is removed from an infant’s diet and replaced by a hypoallergenic formula for three to 5 weeks to determine if allergic symptoms (15, 17). Presumptive diagnosis is then confirmed by an oral challenge using cow’s milk protein. If the patient’s symptoms relapse following the oral cow’s milk protein rechallenge, a diagnosis of CMPA is confirmed. It should be noted that the quality or dynamic of a family’s life, as well as the stress caused by a child’s symptoms on the family, may be affected by the difficulty in clinically diagnosing and treating CMPA (15, 17).

For children that are <1 year old with CMPA, the standard of care includes using hypoallergenic formulas such as extensively hydrolyzed formula or amino acid formula if they are on formula feedings (2, 4, 5, 9, 11, 18). These formulas, respectively, have short peptides and amino acids or amino acids only and are well-tolerated by 90% of children (4, 18–20). Amino acid formulas and extensively hydrolyzed formulas remove allergenicity, which can prevent the immune system from developing tolerance to cow’s milk proteins (13, 21). First-line management of CMPA includes extensively hydrolyzed formula because of its relative cost-efficiency and desirable healthy outcomes (2, 4, 5, 11). However, a small number of infants do not respond well to extensively hydrolyzed formula and their allergy severity requires more stringent CMPA management (2, 4, 5, 11). In such cases, amino acid formulas are frequently used as an alternative (2, 4, 5, 11). Amino acid formulas have been created to address hypersensitivity that may arise from residual proteins in extensively hydrolyzed formulas (13). The use of amino acid formulas for CMPA is a highly debated topic due to the fiscal burden it carries as compared to extensively hydrolyzed formulas. Amino acid formulas, on average, cost 40% more than extensively hydrolyzed formulas in the United States. However, this fiscal cost in the short-term may reduce the longitudinal financial burden untreated or refractory CMPA may cause in diagnostic and further doctors’ appointments (4, 22). Additionally, other hypoallergenic formulas have been recommended, such as rice-based hydrolyzed formulas and soy-based formulas (2). Novel research has also been shown to manipulate infant formula to decrease allergenicity (23, 24) such as the use of enzymes or the use of A2 beta casein.

Short-term symptom improvement studies on amino acid formulas in CMPA are currently limited (25, 26). Parents and physicians prioritize rapid reduction of CMPA symptoms in affected pediatric patients. The purpose of our study was to determine the short-term symptom improvement of a commercial AAF for allergic symptom relief in infants ≤6 months of age with suspected CMPA. We hypothesized that symptoms of CMPA in infants≤6 months of age would decrease in severity at next follow-up visit after initiation of AAF feeding as standard of care for CMPA management.

## 2. Materials and methods

### 2.1. Study objectives

This study is a prospective cohort analysis of de-identified survey data collected from HCPs in the United States using the mobile device application ZSMoments, regarding short-term symptom relief in formula-fed infants <6 of age with suspected CMPA and treated, per standard of care, with AAF.

### 2.2. Study design and participants

Inclusion criteria for HCPs included: greater than 2 years of experience in a clinic-based practice setting, specialization in general pediatrics, pediatric gastroenterology, gastroenterology, or pediatric allergy/immunology; and seeing at least two patients per week who were clinically suspected to have CMPA by their HCP. Exclusion criteria for HCPs included: use of a hypoallergenic formula in less than 2 of their last 10 patients with CMPA, switching patient CMPA management prior to Visit 2, or incomplete data collection.

Patient data was screened for study eligibility in infants 0 to 6 months of age with suspected CMPA by their HCP from July 2021 to October 2021. Data was included for analysis from patients who initiated CMPA management with AAF (Enfamil PurAmino™) and had a follow-up visit 3–6 weeks after. Data were excluded from analysis if AAF feeding was not initiated as CMPA management, patients were older than 6 months at AAF initiation or had incomplete survey responses at either Visit 1 or Visit 2. This study was approved by the Institutional Review Board (IRB) from our institution on 28th April 2021 (IRB 00279920).

### 2.3. Study variables

Pediatric healthcare provider data collected included: gender, specialty, clinical practice setting, years in practice, the average number of patients weekly, and the average number of CMPA patients weekly.

Patient data was collected at screening, Visit 1 and Visit 2 using ZSMoments, a secure mobile-based platform by ZS Associates that allows for the rapid data collection on a user’s mobile device. The initial patient screening included a de-identified questionnaire in which HCPs associated a unique animal with the patient to protect patient health information. During Visit 2, the same HCP used the same animal to access the patient’s file. Patient demographics collected included: age, sex, weight (percentile), height (percentile), family history of allergies, location of the visit, formula used, and symptom severity. Patient data collected at the Visit 2 included: date of Visit 1, method used to assess CMPA status, days since Visit 1, height (percentile), weight (percentile), changes to formula used, symptom severity change, adverse reactions, CMPA symptoms, and parent satisfaction with CMPA management strategy. CMPA management strategies used prior to initiation of AAF were also collected, including antihistamines, probiotics, a maternal elimination diet while breastfeeding, and use of other hypoallergenic formulas.

Gastrointestinal, skin, respiratory, and uncategorized symptoms were collected. Gastrointestinal symptoms included: abdominal pain, diarrhea, nausea, vomiting, regurgitation, burping, flatulence, decreased appetite, hematochezia, mucoid/bloody stools, and constipation. Skin symptoms included: erythema, itching, dry skin, eczema, allergic urticaria, and angioedema. Respiratory symptoms included: nasal obstruction, runny nose, cough, wheezing, laryngeal edema, and shortness of breath. Symptoms classified as “uncategorized” included: watery eyes, conjunctival redness, fussiness, sleep difficulty, pallor, profuse sweating after meals, abnormal growth, and weight gain. Severity of all symptoms were assessed using a 0–3 scale (0, Not Present; 1, Low; 2, Moderate; 3, Severe; or No Score–Not Assessed) at Visit 1 and were re-assessed at Visit 2. Scores from all symptoms were combined at both Visit 1 and Visit 2, yielding a composite score. Overall symptom improvement was defined as a reduction in the composite score between visits. A lack of improvement was defined as a failure to lower overall composite score by Visit 2, either by increased score or maintenance of *status quo*.

### 2.4. Statistical analysis

Continuous study variables were summarized with medians and ranges (minimum/maximum) while categorical study variables were presented as counts and percentages. Data were inspected for completeness and outliers prior to analyses. A complete-case analysis was used to analyze variables with missing data. Statistical significance was evaluated using *t*-tests for the purpose of calculating *p*-values between proportions, with alpha <0.05. All analyses were carried out using SPSS^®^ analytics software (IBM^®^, Armonk, NY, USA).

## 3. Results

### 3.1. Study participants

There were 51 HCPs included in data analysis after nine were excluded for not submitting Visit 2 patient data into the ZSMoments application. HCPs included: general pediatrician (*n* = 45, 87%); pediatric gastroenterologist (*n* = 3, 6%); pediatric allergist/immunologist (*n* = 3, 6%); and gastroenterologist (*n* = 1, 2%). Additionally, HCPs saw varying numbers of patients who were determined clinically in office to have CMPA and were less than 6 months of age per week: 22 (42%) HCPs saw 2–4 patients per week, 20 (38%) saw 5–10 patients per week, nine (17%) saw 11–20 patients per week, and one (2%) saw >20 patients per week. All HCPs (*n* = 51) who prescribed AAF said they would recommend AAF for management of CMPA to HCP colleagues.

**FIGURE 1:**
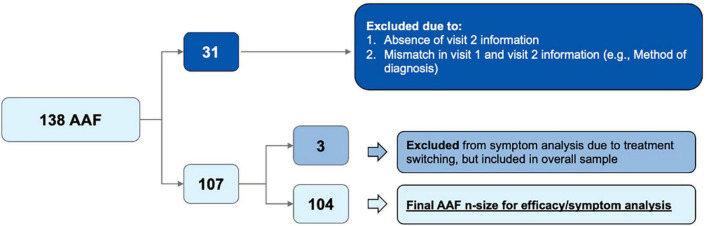
Flow diagram of initial patient population vs. final patient population included in data analysis.

Data from medical records was screened for study eligibility for infants <6 months of age with suspected CMPA (*n* = 404) (Figure 1). Of the 404 infants, 299 (74%) were newly suspected at the first enrollment visit, and 376 (93%) were suspected to have CMPA via clinical assessment.

Further, a total of 138 patients who initiated AAF based on clinician assessment were identified. Of this group, 31 patients were excluded due to missing data, mismatched data between Visit 1 and Visit 2, or poor-quality survey data (*n* = 31). The final cohort (*n* = 107) included 104 patients who remained in the AAF cohort and three patients who had a treatment change between Visit 1 and Visit 2 and therefore were excluded from the final symptom severity analysis. Patient demographics is depicted below in Table 1. Of the 107 patients in the final sample, CMPA assessments were made through clinical assessment (*n* = 97), family history (*n* = 51), elimination diet (*n* = 67), oral re-challenge (*n* = 3), food panel testing (*n* = 5), skin prick testing (*n* = 9), IgE serum assay (*n* = 6), and atopic patch testing (*n* = 5). Additionally, most CMPA suspected assessments were made by pediatricians (*n* = 88).

**TABLE 1:** Patient demographics.

Gender, *n* (%)	
Male	42 (40%)
Female	62 (60%)
**Age at enrollment (Months)**	
Median	3
Mean	3.3
Allergy History, *n* (%) Family History of Atopy	49 (46%)
Method of CMPA Diagnosis, *n* (%)	97 (91%)
**Clinical assessment**	
Elimination Diet	67 (63%)
Family History	51 (48%)
Skin Prick Test	9 (8%)
Food Panel Test	5 (5%)
IgE Serum Assay	6 (6%)
Atopic Patch Test	5 (5%)
Food Antigen Re-challenge	3 (3%)
Prior CMPA Management Strategies, *n* (%) Other AAF	3 (3%)
Probiotics	36 (34%)
Breastfeeding	19 (18%)
Antihistamines	18 (17%)
**Duration (Weeks) between Visit 1 and Visit 2, n (%)**	
3 weeks	7 (7%)
4 weeks	19 (18%)
5 weeks	41 (38%)
> 5 weeks	40 (37%)

### 3.2. Gastrointestinal, skin, and respiratory symptoms

**FIGURE 2:**
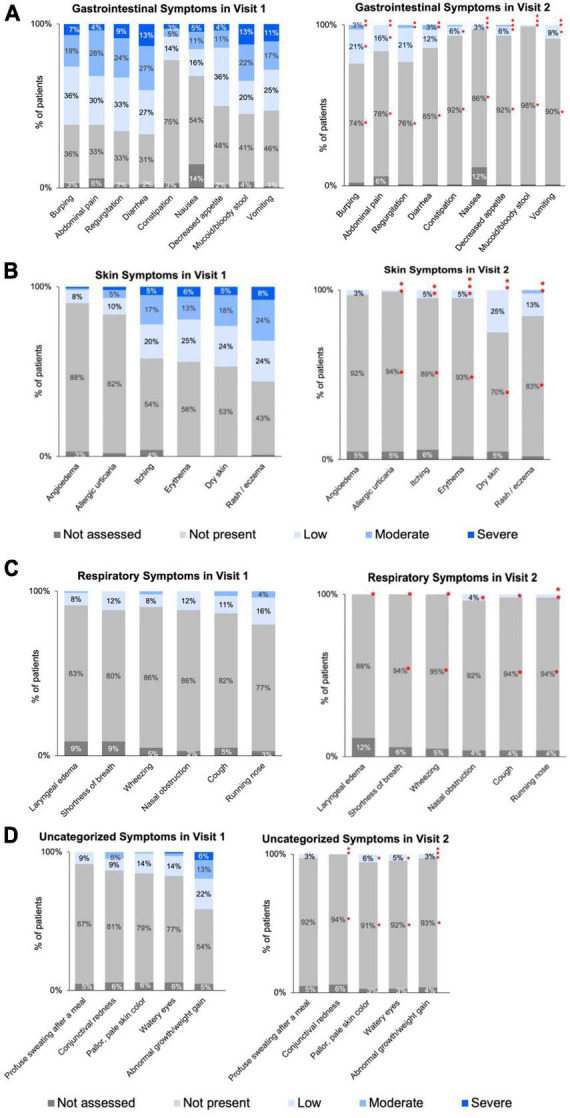
**(A)** Visit 1 to Visit 2: severity of gastrointestinal symptoms for infants ≤6 months of age with diagnosed or suspected CMPA. **(B)** Distribution of skin symptoms severity across two visits for infants ≤6 months of age with diagnosed or suspected CMPA. **(C)** Distribution of respiratory symptoms severity across two visits for infants ≤6 months of age with diagnosed or suspected CMPA. **(D)** Distribution of uncategorized symptoms severity across two visits for infants ≤6 months of age with diagnosed or suspected CMPA. *Denotes statistically significant (*p* ≤ 0.05) decrease in symptom severity between Visit 1 and Visit 2. Specific *p*-values listed in Supplementary Tables 1–4. Asterisks may appear stacked on top of each other if the higher symptom severity level is no longer visible as there were no/few patients with that symptom severity.

Gastrointestinal, skin, respiratory, and uncategorized symptom severity data categorized and collected by HCPs at Visit 1 and Visit 2 are summarized in Figures 2A–D, and in Supplementary Tables 1–4. Of 104 patients, the percentage of patients with improvement in symptoms between Visit 1 and Visit 2 is also summarized in Table 2. In addition, patients receiving AAF for CMPA management demonstrated overall average improvements in gastrointestinal symptoms (94%), skin symptoms (87%), respiratory symptoms (86%), and uncategorized symptoms (89%). Symptom improvement was consistent across different durations between Visit 1 and Visit 2.

**TABLE 2:** Distribution of gastrointestinal, skin, respiratory, and uncategorized symptoms in infants ≤6 months of age with suspected CMPA.

Symptoms	Visit 1, n^1^	Visit 2, *n*	Improvement,%^2^
**Gastrointestinal**			
Diarrhea	73	66	90%
Regurgitation	70	56	80%
Abdominal pain	66	56	85%
Burping	67	52	78%
Vomiting	58	54	93%
Mucoid/bloody stool	57	56	98%
Decreased appetite	54	49	91%
Nausea	34	33	97%
Constipation	27	21	78%*
**Skin**			
Rash/eczema	57	52	91%
Dry Skin	52	39	75%
Erythema	45	43	96%
Itching	43	41	95%
Allergic urticaria	16	15	94%*
Angioedema Respiratory	11	9	82%*
Running nose	20	20	100%*
Cough	15	13	87%*
Nasal obstruction	12	10	83%*
Wheezing	9	9	100%*
Shortness of breath	12	12	100%*
Laryngeal edema	8	8	100%*
**Uncategorized**			
Abnormal growth/weight gain	43	42	98%
Watery Eyes	18	15	83%*
Pallor, pale color skin	17	12	71%*
Conjunctival redness	13	13	100%*
Postprandial diaphoresis	10	7	70%*

^1^Number of patients who presented with a given CMPA symptom at the first visit and were assessed at the second visit.

^2^Only patients with a given CMPA symptom present at the first visit were included in the percent improvement calculation.

*n < 30.

## 4. Discussion

In this prospective cohort analysis, we evaluated changes in short-term symptom severity in formula-fed infants <6 months of age with suspected CMPA and treated with AAF through de-identified survey data collected from HCP utilizing a secure mobile application device. This is the largest evaluation of the short-term symptom improvement conducted in the United States of AAF use in infants with clinically suspected CMPA by their HCP. To address this knowledge gap, we report a cohort of 107 infants <6 months of age who presented with CMPA symptoms to their HCP and initiated AAF treatment in this analysis. While amino acid formulas have been proven to be effective over the long term, parents and healthcare professionals are concerned with quickly improving short-term symptoms (4, 15, 27). Additionally, previous cohort studies from Poland and France have reported on this issue (28). However, there is still a lack of research on the short-term symptom resolution of cow’s milk protein allergy (CMPA).

In the current health care provider survey, HCPs were able to assess symptom severity change in the clinical setting and assess the short-term symptom improvement of CMPA symptoms with AAF by utilizing the ZSMoments data collection application to document real-time patient data accurately and freely. ZSMoments, by ZS Associates, is a mobile-based application platform that allows for the rapid, secure data collection on a user’s mobile device. Most patients experienced gastrointestinal, skin, and other common symptoms of CMPA. Additionally, a smaller percentage of patients experienced respiratory symptoms. Total improvement in allergic symptoms remained consistent across gastrointestinal, skin, respiratory, and uncategorized symptoms. Most patients experienced an improvement in GI, skin, and respiratory symptoms when receiving an AAF for CMPA symptoms by the next follow-up visit.

Cow’s milk protein allergy is an allergic reaction to a cow’s milk protein which may be found in a mother’s breast milk or in cow’s milk (3). When an infant consumes cow’s milk protein, their immune system mistakes the protein as a foreign invader and will produce an allergic reaction against the protein (3). Therefore, symptoms can vary depending on the type of allergic reaction (immediate or delayed) and where the allergic reaction is produced (skin or gastrointestinal tract) (2–4, 9–11). CMPA mainly affects infants younger than one year old and is typically outgrown by the ages of three to five. Approximately 50% of children resolve CMPA by the age of one, and 80 to 90% resolve CMPA by the age of 5 years old (5).

The inclusion of infants ≤6 months of age was based upon HCP assessment of CMPA symptoms. In clinical practice, CMPA is diagnosed by: an allergy-focused history, symptom resolution following a maternal cow’s milk elimination diet, and the reemergence of symptoms in infants using an oral challenge test after other abnormalities such as gastrointestinal infection are ruled out (2, 5, 9, 15). CMPA may be challenging to diagnose because of non-specific symptoms such as regurgitation, abdominal pain, and feeding difficulties which may resemble GERD and functional disorders of infancy (2, 5, 10, 15). The IgE subtype of CMPA presents as an immediate allergic reaction and can therefore be diagnosed using serology and skin prick test (2, 5, 10, 15). However, the non-IgE subtype typically presents as a delayed immunological reaction in which no specific laboratory test can be used to confirm the diagnosis (2, 5, 10, 15). Although the majority of patients were diagnosed with CMPA through the elimination diet, only a small fraction of patients who utilized the elimination diet continuing to the oral re-challenge despite the elimination-oral challenge being the gold standard to diagnose CMPA. Parents are typically apprehensive to re-expose their infant to cow’s milk after symptom resolution in fear of symptom reemergence making it difficult to formally diagnose the sentence in a clinical setting (2, 5, 10, 15).

Extensively hydrolyzed formulas are a hypoallergenic formula used to manage symptoms of CMPA because they increase tolerance to cow’s milk protein allowing infants to return to cow’s milk products more quickly (2, 4, 9, 29). According to the American Academy of Pediatrics, extensively hydrolyzed formulas are the first-line hypoallergenic formula used to manage CMPA due to high efficacy and low cost. Extensively hydrolyzed formulas are produced using enzyme hydrolysis, heat-induced thermal hydrolysis, and ultra-filtration of cow’s milk (11). Therefore, specific cow’s milk-derived peptides in extensively hydrolyzed formulas may continue to cause CMPA symptoms in children with moderate to severe CMPA (30). Amino acid formulas are used as a second-line hypoallergenic formula option when extensively hydrolyzed formula is discontinued because of feeding difficulties, development of allergic reaction to extensively hydrolyzed formula, or lack of symptom improvement (15). Amino acid formulas have free amino acids as the nitrogen source, therefore avoiding exposure to cow’s milk proteins (11, 31). Alternatively, amino acid formulas are used as the first-line hypoallergenic management when infants have severe symptoms of CMPA, which include: anaphylaxis, food protein-induced enterocolitis syndrome, allergic eosinophilic esophagitis, milk-induced chronic pulmonary disease, failure to thrive, and multi-organ system symptoms (4, 22).

Other hypoallergenic treatment alternatives include milk from other mammals, such as sheep; however, this is contraindicated in infants with CMPA because these animals have the highest levels of cross-over allergenicity due to their intact proteins (2). Alternatively, rice-based hydrolyzed formulas are well-tolerated by infants and are better-tasting compared to extensively hydrolyzed formulas, but studies suggest that a minority of infants show a weakly positive immunological skin prick test (2). Soy-based formulas may be used; however, the American Academy of Pediatrics suggests that 10–14% of patients with CMPA will develop a soy allergy, especially in infants with the IgE subtype of CMPA (2).

Recently, there have been novel approaches being made to augment infant formula to improve digestibility and reduce allergenicity for the use in management of CMPA symptoms. One example of this includes the addition of endo-β-N-acetylglucosaminidases from bifidobacterium longum subspecies to infant formulas (23). This enzyme can be used to release *N*-glycans to increase the bifidogenicity of infant formula as N-glycans have a high similarity to human milk oligosaccharides which stimulate development of a *Bifidobacterium*-rich microbiome physiologically (23). Another example includes infant formula made by A2 beta caseins which is a novel approach used to avoid A1 beta casein (24).

Additionally, some suggest using amino acid formulas as first-line management for severe cases of CMPA (including complex disease presentations, multiple food involvement, or when extensively hydrolyzed formulas fail to reduce burden of symptoms) due to the higher cost and the recent increase in CMPA in children (4, 22). Studies also have suggested that, although amino acid formulas are generally more expensive when compared to extensively hydrolyzed formula, this difference in cost may be offset in the long-term because patients may have fewer hospitalizations due to CMPA. In Turkey, for example, CMPA management using an amino acid formula provided economic benefits, improved tolerability, and better clinical outcomes due to their ability to treat mild to severe CMPA and avoid allergic reactions to cow’s milk peptides found in extensively hydrolyzed formulas (11). Based on the findings of this study, it appears that the early introduction of amino acid formula in a clinical setting may decrease symptom severity of both gastrointestinal and systemic allergy symptoms in infants with CMPA. However, decision-making regarding using amino acid formula should consider factors such as cost and the potential benefits of early tolerance induction with an extensively hydrolyzed formula (32). It should be noted that further research is needed to confirm these findings and determine the long-term effects of early amino acid formula introduction in infants with CMPA.

There are several limitations to this study. This study lacked a control group, limiting the ability to determine if symptoms improved spontaneously or due to initiation of a CMPA management strategy. It is possible that some symptoms reported by parents in infants with suspected CMPA may be functional and could improve spontaneously in time. Additionally, there was a small sample size of infants experiencing skin respiratory, and uncategorized symptoms. Further, many patients were suspected clinically to have CMPA by their HCP alone and did not receive documentation of allergy by food antigen re-challenge or IgE testing. IgE assays can be beneficial in IgE-mediated food allergy but are not useful in diagnosing non-IgE CMPA. Finally, due to the short-term focus of these analyses, patients were only evaluated at the first follow-up visit, preventing researchers from ensuring the persistence of symptom resolution in the long term.

## 5. Conclusion

Our study is the largest prospective survey examining short-term changes in symptom severity in infants <6 months of age managed with AAF for suspected CMPA, and the first conducted in the United States. We evaluated 26 symptoms of CMPA across four categories: gastrointestinal, skin, respiratory, and uncategorized symptoms. Our study found that infants with CMPA who received AAF had statistically significant decreases in symptom severity for gastrointestinal (94%), skin (87%), respiratory (86%), and uncategorized symptoms (89%). To our knowledge, this is the first study to assess this diverse array of CMPA symptoms. Our study suggests that AAF may be an effective option for CMPA management for infants 6 months of age and under with suspected CMPA, often by the next follow-up visit. However, cost-effectiveness and the potential benefit of early tolerance induction with extensively hydrolyzed formulas should be considered. Further randomized controlled with larger cohorts are needed to confirm these preliminary results.

## Data availability statement

The raw data supporting the conclusions of this article will be made available by the authors, without undue reservation.

## Ethics statement

The studies involving human participants were reviewed and approved by the Johns Hopkins All Children’s Hospital Institutional Review Board. Written informed consent to participate in this study was provided by the participants’ legal guardian/next of kin.

## Author contributions

MW and JV: conceptualization, investigation, methodology, and supervision. JVB, LL, JB, JF, and JMB: data curation and formal analysis. JVB and LL: writing–original draft. MW, JVB, LL, JB, JF, LO, PS, JMB, and JV: writing–review and editing. All authors contributed to the article and approved the submitted version.
