# Supplementary material for: Short-term symptom improvement in infants with suspected cow’s milk protein allergy using amino acid formula: a prospective cohort analysis
Source: Front Nutr. 2023 Jun 20;10:1208334. doi: 10.3389/fnut.2023.1208334 (PMC10318537; doi:10.3389/fnut.2023.1208334)
Supplement: Supplementary file 1 [file Table_1.DOCX]

Supplementary Material

# Supplementary Data

|  | Not assessed | Not present | Low | Moderate | Severe |
| --- | --- | --- | --- | --- | --- |
| Burping | 0.653 | 0.000 | 0.021 | 0.000 | 0.007 |
| Abdominal pain | 1.000 | 0.000 | 0.021 | 0.000 | 0.044 |
| Regurgitation | 0.563 | 0.000 | 0.061 | 0.000 | 0.002 |
| Diarrhea | 0.563 | 0.000 | 0.005 | 0.000 | 0.000 |
| Constipation | 0.315 | 0.001 | 0.038 | 0.098 | 0.082 |
| Nausea | 0.538 | 0.000 | 0.001 | 0.001 | 0.024 |
| Decreased appetite | 0.563 | 0.000 | 0.000 | 0.003 | 0.044 |
| Mucoid/bloody stools | 0.176 | 0.000 | 0.000 | 0.000 | 0.000 |
| Vomiting | 1.000 | 0.000 | 0.002 | 0.000 | 0.001 |

**Supplementary Figure 1.** Visit 1 to Visit 2 improvement in severity of gastrointestinal symptoms for infants <6 months of age with suspected CMPA.

|  | Not assessed | Not present | Low | Moderate | Severe |
| --- | --- | --- | --- | --- | --- |
| Angioedema | 0.473 | 0.252 | 0.123 | 0.318 | 0.318 |
| Allergic urticaria | 0.251 | 0.005 | 0.005 | 0.024 | 0.157 |
| Itching | 0.519 | 0.000 | 0.001 | 0.000 | 0.024 |
| Erythema | 0.157 | 0.000 | 0.000 | 0.000 | 0.013 |
| Dry skin | 0.024 | 0.010 | 0.873 | 0.000 | 0.024 |
| Rash / eczema | 0.563 | 0.000 | 0.051 | 0.000 | 0.004 |

**Supplementary Figure 2.** Visit 1 to Visit 2 improvement in severity of skin symptoms for infants <6 months of age with suspected CMPA.

|  | Not assessed | Not present | Low | Moderate | Severe |
| --- | --- | --- | --- | --- | --- |
| Laryngeal edema | 0.492 | 0.238 | 0.004 | 0.318 | - |
| Shortness of breath | 0.424 | 0.002 | 0.000 | - | - |
| Wheezing | 1.000 | 0.019 | 0.004 | 0.157 | - |
| Nasal obstruction | 0.702 | 0.123 | 0.038 | - | - |
| Cough | 0.735 | 0.005 | 0.010 | 0.082 | - |
| Running nose | 0.702 | 0.000 | 0.000 | 0.044 | - |

**Supplementary Figure 3.** Visit 1 to Visit 2 improvement in severity of respiratory symptoms for infants <6 months of age with suspected CMPA.

|  | Not assessed | Not present | Low | Moderate | Severe |
| --- | --- | --- | --- | --- | --- |
| Profuse sweating after a meal | 1.000 | 0.178 | 0.075 | - | - |
| Conjunctival redness | 1.000 | 0.003 | 0.002 | 0.024 | - |
| Pallor, pale skin color | 0.309 | 0.011 | 0.038 | 0.318 | - |
| Watery eyes | 0.309 | 0.002 | 0.019 | 0.157 | 0.318 |
| Abnormal growth/weight gain | 0.735 | 0.000 | 0.000 | 0.000 | 0.013 |

**Supplementary Figure 4.** Visit 1 to Visit 2 improvement in severity of uncategorized symptoms for infants <6 months of age with suspected CMPA.
